# Comparison of Early Postoperative Diaphragm Muscle Function after Lobectomy via VATS and Open Thoracotomy: A Sonographic Study

**DOI:** 10.3390/life14040487

**Published:** 2024-04-09

**Authors:** Janusz Kocjan, Mateusz Rydel, Damian Czyżewski, Mariusz Adamek

**Affiliations:** 1 Department of Thoracic Surgery, Faculty of Medicine with Dentistry Division, Medical University of Silesia, 40-055 Katowice, Poland; mateusz.rydel@wp.pl (M.R.); damianczyzewski@o2.pl (D.C.); m.adamek@e.pl (M.A.); 2 Department of Radiology, Faculty of Health Sciences with Institute of Maritime and Tropical Medicine, Medical University of Gdansk, 80-210 Gdansk, Poland

**Keywords:** diaphragm, VATS, thoracotomy, thoracic surgery, lung cancer

## Abstract

Although a growing body of evidence emphasizes the superiority of VATS over conventional thoracotomy, little is still known about early postoperative diaphragm muscle function after lobectomy via these two approaches. To fill the gap in existing literature, we conducted a comparative study between VATS and conventional thoracotomy in terms of postoperative diaphragm muscle function, assessing its contractility, strength, the magnitude of effort and potential risk of dysfunction such as atrophy and paralysis. A total of 59 patients (30 after VATS), who underwent anatomical pulmonary resection at our institution, were enrolled in this study. The control group consisted of 28 health subjects without medical conditions that could contribute to diaphragm dysfunction. Diaphragm muscle was assessed before and after surgery using ultrasonography. We found that both surgical approaches were associated with postoperative impairment of diaphragm muscle function—compared to baseline data. Postoperative reduction in diaphragm contraction was demonstrated in most of the 59 patients. In the case of the control group, the differences between measurements were not observed. We noted that lobectomy via thoracotomy was linked with a greater percentage of patients with diaphragm paralysis and/or atrophy than VATS. Similar findings were observed in referring to diaphragm magnitude effort, as well as diaphragm contraction strength, where minimally invasive surgery was associated with better diaphragm function parameters—in comparison to thoracotomy. Disturbance of diaphragm work was reported both at the operated and non-operated side. Upper-right and left lobectomy were connected with greater diaphragm function impairment than other segments. In conclusion, the VATS technique seems to be less invasive than conventional thoracotomy providing a better postoperative function of the main respiratory muscle.

## 1. Introduction

Lung cancer is one of the leading causes of mortality among women and men, accounting for over 1.8 million deaths worldwide per year [1]. Treatment options and recommendations depend on cancer type, clinical stage and patient-specific factors, and may include surgery, chemotherapy, radiation, targeted therapy, as well as a combination of these methods [2].

Pulmonary lobectomy is the most frequent surgical procedure for operable lung cancer. The method of lobe resection is mainly considered between two approaches: conventional open thoracotomy and video-assisted thoracoscopic surgery (VATS). In recent years, most studies demonstrated that minimal invasive lobectomy has substantial advantages over standard thoracotomy for early-stage non-small cell lung cancer regarding its safety, feasibility, better quality of life, faster time to return to work, as well as lower mortality rates, less blood loss during operation, decrease in postoperative pain, reduced analgesic intake, less postoperative shoulder dysfunction, shorter length of hospital stay and chest tube drainage [3,4,5,6,7,8,9,10,11]. However, despite a growing body of evidence it is still not known whether VATS, due to its minimally invasive approach, allows the preservation of better postoperative diaphragm muscle function compared to open thoracotomy. Many previous studies that investigated the diaphragm after pulmonary resection indicate numerous postoperative disorders including a reduction in active diaphragmatic contractility [12], a decrease in maximal transdiaphragmatic pressure [13] and an increase in intercostal and accessory breathing muscle recruitment [14], but the vast majority of this research was conducted on either VATS or thoracotomy populations. In fact, only three papers compared these two approaches in the range of this main breathing muscle working [15,16,17]. However, these items have some limitations and have not fully explored this issue. Given the above, we conducted this study to fill the gap in the existing literature.

The purpose of the present study was to comprehensively assess the diaphragm muscle function parameters at the early postoperative stage among patients who have undergone lobectomy via video-assisted thoracoscopic surgery or standard thoracotomy, as well as to investigate the frequency of occurrence of the postoperative diaphragm muscle dysfunction in each of the two approaches for early-stage non-small cell lung cancer. 

## 2. Materials and Methods

### 2.1. Participants Selection Process and Eligibility Criteria

Our study included two groups of individuals. The first group (clinical) contained 59 adults diagnosed with resectable lung cancer pathologically confirmed, who were treated surgically. Of these, 50.84% (n = 30) had undergone lung resection via video-assisted thoracoscopic surgery (VATS) and the remaining 49.16% (n = 29) had undergone open thoracotomy (OT). Patients who had the following features were excluded from the study: previous thoracic and/or abdominal surgery, concomitant diseases that can impair diaphragm function (stroke, multiple sclerosis, Guillan–Barre syndrome, myasthenia gravis, muscular dystrophy, asthma, polyneuropathy), poor ultrasound view of diaphragm muscle and the patient’s inability to understand verbal instructions about breathing maneuvers. Cases with segmentectomy and bilobectomy were also denied. Demographics and preoperative information such as age, height, weight, gender, involved side and resected lobe were collected from the medical records of the patients.

The control group consisted of 28 adult volunteers. A subject data sheet was used to collect demographic data, such as age, sex, weight, height and medical status. The exclusion criteria for this group were as follows: history of thoracic and/or abdominal surgery and presence of chronic disease that can disturb diaphragm muscle work.

### 2.2. Surgical Techniques

Surgical treatment of NSCLC involved anatomical resection of the lung lobe where the tumor was located (lobectomy) and removal of mediastinal lymph nodes (lymphadenectomy). All patients were operated on by well-trained surgeons with extensive experience who worked at the Department of Thoracic Surgery. The surgical approach was non-randomly chosen between a lateral thoracotomy approach or a minimally invasive method using videothoracoscopy by the surgeon team performing the lobectomy, based on the clinical picture and attributes such as tumor size, patient age, pulmonary function and the patient’s general condition. 

Both procedures were carried out according to a standardized scheme. The classic method was used for access through an anterolateral thoracotomy, and after the procedure, two drains were placed in the pleural cavity through separate 2–3 cm incisions. The minimally invasive method was performed using the uniportal technique, i.e., through one incision about 4 cm long, into which one drain was inserted into the pleural cavity. Each procedure was performed under general anesthesia (Propofol, Fentanyl, Sewofluran). Patients were intubated with a double-lumen tube and selectively ventilated to the lung on the side opposite to the operated one. Post-surgery pain management was primarily achieved by oxycodone from an infusion pump supplemented with per os nonsteroidal anti-inflammatory drugs and paracetamol.

### 2.3. Diaphragm Muscle Ultrasound Assessment

Sonographic diaphragm muscle imaging was carried out using an Aloka Prosound Alpha 10 ultrasound machine. Patients were examined in the supine position, which prevents any paradoxical movement, limits compensatory active expiration by the anterior abdominal wall which may mask paralysis, provides less variability and reduces side-to-side variations. The high-frequency (>10 MHz) linear array transducer was placed at the anterior axillary line in the area named Zone of Apposition and positioned to obtain a sagittal image at the intercostal space between the 8th and 10th ribs. In a two-dimensional B-Mode set, the right and left hemidiaphragms were visualized through the liver and spleen window, respectively, as a three-layer structure with two outer echogenic layers of pleura and peritoneum lining an inner hipoechoic layer of muscle. During the inspiratory and expiratory phases, this area normally thickens and shortens, respectively [18,19].

Before measurements, all participants were practically instructed about breathing maneuvers to perform. First, the subjects were examined during the spontaneous respiration phase to identify the movement of a diaphragm. In the next stage, the patient was asked to perform a maximum inspiration and maximum forced expiration. The distance between the echogenic lines that determines inspiratory (ThIns) and expiratory (ThExp) thickness was measured in frozen images. For each breathing maneuver, three readings were taken and the average values were finally included in statistical analysis. All participants were scanned two times by the same specialist with extensive experience. In the clinical group, measurements were performed the day before the surgery and 3–5 days after surgery (on the first day after the drains were removed). In the case of the control group, the second measurement took place 3–6 days after the first measurement.

The following diaphragm muscle function indices were calculated from obtained inspiratory and expiratory thickness [18,19]:

1. DTF (Diaphragm Thickness Fraction), reflecting the magnitude of diaphragm effort. We used the following standardized formula: DTF = (ThIns − ThExp)/ThExp × 100%. The normal percentage for the supine position is more or equal to 65%. DTF values less than 20% are consistent with diaphragm muscle paralysis.
2. DTR (Diaphragm Thickening Ratio), reflecting the diaphragm muscle strength. This index was calculated using the following formula: DTR = ThIns/ThExp. Higher values represent a better outcome. The normal value is between 1.7 to 2.0.
3. Δ (Delta)—differences between preoperative (pre) and postoperative (post) values on each side were calculated using the following formulas: ΔThIns = ThIns (pre) − ThIns (post), ΔThExp = ThExp (pre) − ThExp (post), ΔDTF = DTF (pre) − DTF (post) and ΔDTR = DTR (pre) − DTR (post). For the precise comparative analysis, the differences between preoperative and postoperative values were also expressed as a percent of the preoperative amplitude: ΔThIns (%) = ΔThIns × 100/ThIns (pre), ΔThExp (%) = ΔThExp × 100/ThExp (pre), ΔDTF (%) = ΔDTF × 100/DTF (pre) and ΔDTR (%) = ΔDTR × 100/DTR (pre).
4. Side-to-side variability (stsv)—differences between the left and right hemidiaphragm were calculated using the following formulas: ThIns (StSv) = ThIns (left) − ThIns (right), ThExp (StSv) = ThExp (left) − ThExp (right), DTF (StSv) = DTF (left) − DTF (right) and DTR (StSv) = DTR (left) − DTR (right). The obtained results are given as absolute values.

### 2.4. Statistical Analysis

Statistical analysis was performed using PQ STAT software. The data distribution was tested using the Kolmogorov–Smirnov test. Quantitative data were expressed as mean (M), standard deviation (SD) and 95% Confidence of Interval (CI), while quantitative and categorical variables were presented as numbers and percentages. One-way analysis of variance (ANOVA) and *t*-student test were accomplished to detect differences between groups. The Chi-square test was used to check differences between proportions. To compare preoperative and postoperative data, the Wilcoxon signed rank test was chosen. For all analyses, a *p*-value less than 0.05 was considered statistically significant.

## 3. Results

**Figure 1:**
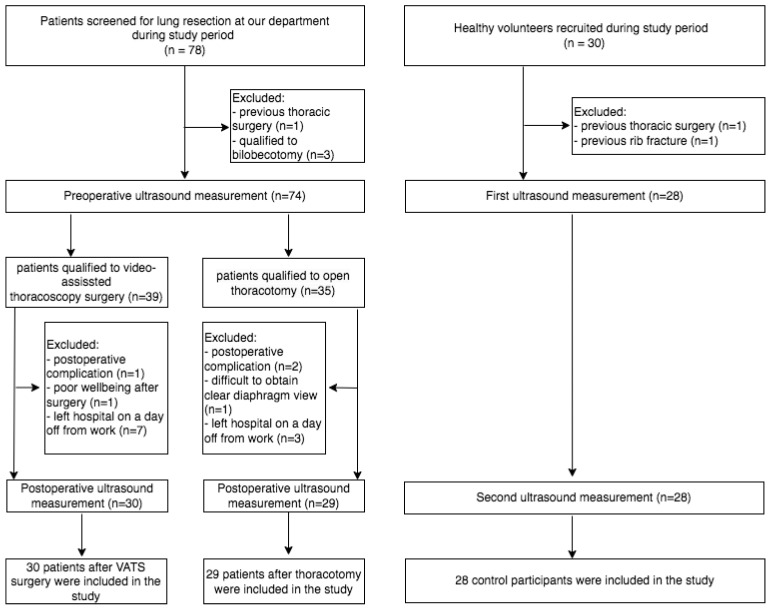
Schematic diagram of patient selection.

A flow chart for study participants is presented in Figure 1.

There was no conversion to thoracotomy among patients who initially underwent VATS lobectomy. All three groups were comparable with respect to the number of participants, mean age and gender composition. Both clinical groups were also homogeneous in terms of involved side and lobe resection. Detailed baseline characteristics are given in Table 1.

There were no differences between the three examined groups in relation to baseline parameters of the diaphragm function on the left (ThIns: (left) = 0.271, (right) = 0.291; ThExp: (left) = 0.356, (right) = 0.364, DTF: (left) = 0.980, (right) = 0.923; DTR: (left) = 0.996, (right) = 0.915) and right (ThIns: (left) = 0.176, (right) = 0.415; ThExp: (left) = 0.795, (right) = 0.741 DTF: (left) = 0.131, (right) = 0.320 DTR: (left) = 0.158, (right) = 0.313) sides of the body. We demonstrated a significant both-sided postoperative decrease of inspiratory thickness, diaphragm thickness fraction and diaphragm thickening ratio compared to preoperative values, regardless of the surgical approach and side of operation. However, a greater percentage of deterioration of the above-mentioned parameters was noted among patients who underwent open thoracotomy rather than VATS (Table 2 and Table 3). In both clinical groups, most of the patients had worse postoperative values of each analyzed diaphragm parameter, but at this point, statistical significance between VATS and OT groups was not found. 

In the control group, the differences between first and second measurements in case of an inspiratory thickness (left side: 3.19 ± 0.45 vs. 3.20 ± 0.49, *p* < 0.822; right side: 3.16 ± 0.43 vs. 3.18 ± 0.45, *p* < 0.697), expiratory thickness (left side: 2.26 ± 0.59 vs. 2.28 ± 0.61, *p* < 0.746; right side: 2.23 ± 0.62 vs. 2.22 ± 0.61, *p* < 0.773), thickness fraction (left side: 39.62 ± 17.86. vs. 40.15 ± 19.57, *p* < 0.809; right side: 38.22 ± 16.08 vs. 38.74 ± 17.44, *p* < 0.811) and thickening ratio (left side: 1.62 ± 0.23 vs. 1.64 ± 0.21, *p* < 0.776; right side: 1.60 ± 0.22 vs. 1.61 ± 0.20, *p* < 0.001) were not observed. However, the presented values of diaphragm parameters obtained in the second measurement were significantly higher in the non-operated group—compared to post-surgery values measured in VATS (all *p* < 0.001) and OT (all *p* < 0.001) groups, respectively. In this group, only one of the participants had worse outcomes, while 39.2%, 25%, 35.7%, and 42.8% had slightly better values of inspiratory and expiratory thickness, as well as DTF and DTR indexes, respectively.

**Figure 2:**
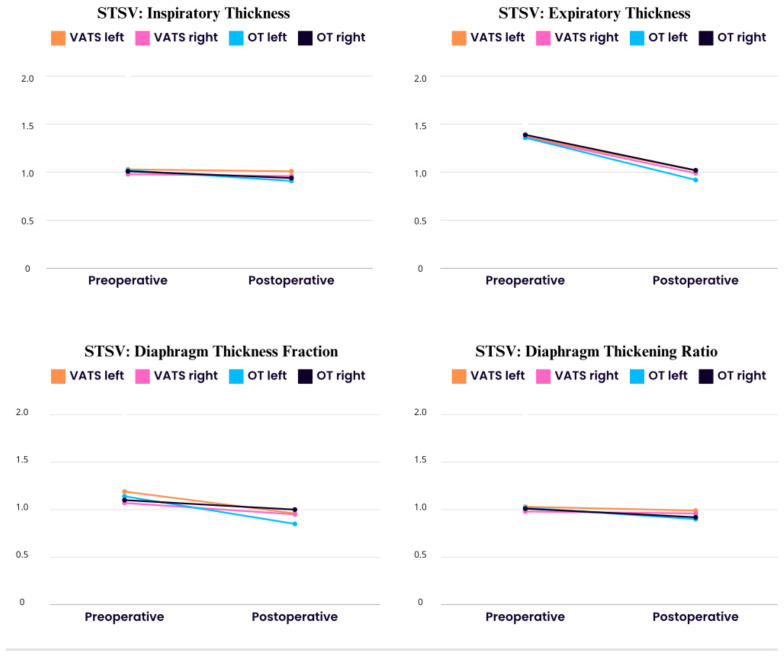
Preoperative and postoperative differences between hemidiaphragms.

In Figure 2, we presented values of the stsv (side-to-side variability) index, which measures differences between the left and right hemidiaphragm. We demonstrated a postoperative decrease compared to preoperative calculation in both groups in all parameters, which indicates dysfunction after lobectomy on both diaphragm cupolaes. 

**Figure 3:**
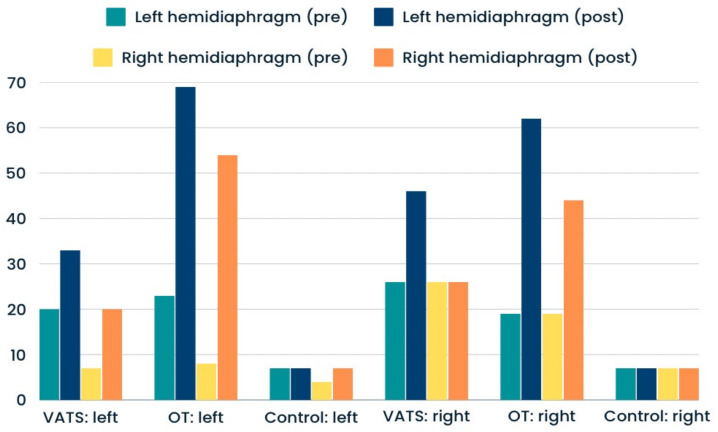
Percentage of preoperative (pre) and postoperative (post) hemidiaphragm paralysis in analyzed groups.

**Figure 4:**
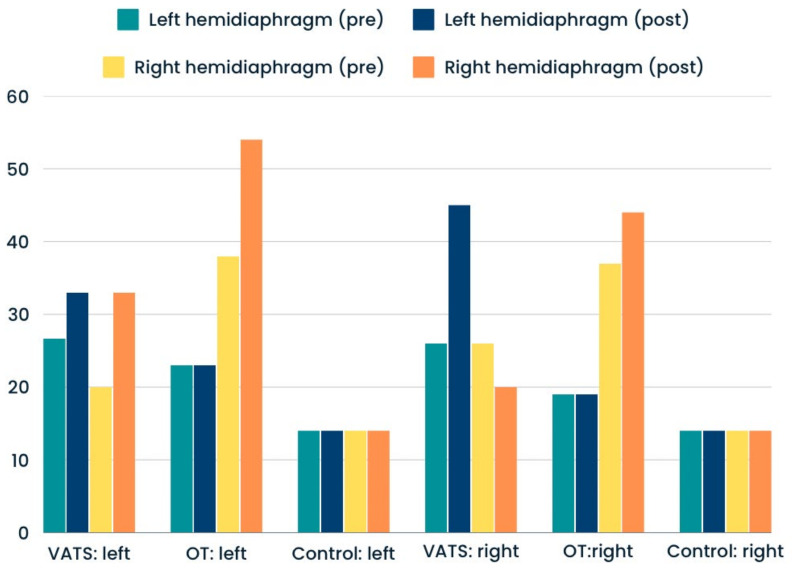
Percentage of preoperative (pre) and postoperative (post) hemidiaphragm atrophy in analyzed groups.

**Figure 5:**
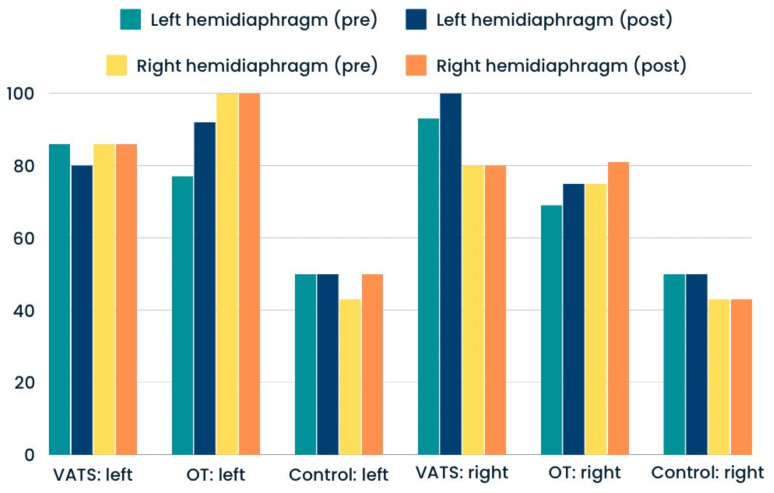
Percentage of preoperative (pre) and postoperative (post) hemidiaphragm weakness in analyzed groups.

Significantly greater percentages of hemidiaphragm paralysis were found among patients who underwent open thoracotomy rather than VATS. This type of dysfunction was present in similar numbers at the operated and non-operated sides (Figure 3). The proportions of atrophy and weakness were only slightly higher after surgery in both groups—compared with input data (Figure 4 and Figure 5). In the case of the control group, we reported all three types of diaphragm dysfunctions on a similar level at the first and second measurements (Figure 3, Figure 4 and Figure 5).

Values of the delta (Δ) index for diaphragm parameters divided into areas of lung resection are given in Table 4. Due to the small sample size, patients with middle lobe resection were missed in the analysis. Our findings showed each lobe resection was associated with impairment of analyzed parameters. The greatest postoperative diaphragm deterioration was identified in patients after upper-left and right lobe resection—compared to lower lobes, but statistical significance was observed only in a few parameters. In all cases, slightly higher values were observed on the operated rather than the non-operated side, but the level of differences was not significant (Table 4).

## 4. Discussion

Analysis of the data presented in Table 2 and Table 3 indicates that both surgical procedures were significantly associated with a decrease in postoperative inspiratory diaphragm thickness, which was not observed in the case of the control group. Similarly, the values of diaphragm thickening ratio (DTR) reflecting diaphragm muscle strength were reduced—compared to baseline data. Taking into consideration the Delta Index, video-assisted thoracoscopic surgery had a less detrimental impact on the above-mentioned parameters than open thoracotomy. Our findings are consistent with previous studies. Bernard [20] and Nomori [21] also showed that conventional thoracotomy was associated with a higher decrease in diaphragm muscle strength measured as Maximal Inspiratory Pressure (MIP) 2 days and 1 week after surgery, respectively. In contrast, Brocki [22] et al. found that respiratory muscle strength was not affected postoperatively, but in this study, second measurements were performed only 2 weeks after surgery.

The Diaphragm Thickness Fraction (DTF) reflecting the magnitude of diaphragm effort was reduced more after open thoracotomy. Furthermore, the percentage of postoperative diaphragm paralysis, detected as DTF < 20%, was also higher in this group. The mean values of postoperative expiratory diaphragm thickness were close to the initial data. This finding allows us to conclude that the reduction in the DTF index is primarily associated with a decrease in inspiratory thickness rather than a decrease in both parameters. No differences between groups were found in the percentage of diaphragm atrophies diagnosed as expiratory thickness less than 2 mm. To date, there are no previous studies comparing the DTF index and expiratory thickness between OT and VATS, but one paper evaluated the impact of the surgery approach on diaphragm contractility by measuring its excursion. Spadaro et al. [16] demonstrated that patients after VATS were characterized by better diaphragm excursion in the first 24 h after surgery. However, the measurements were performed only during spontaneous breathing, while the DTF index refers to maximal inspiration.

All of the abnormalities we described here occurred on both the operated and non-operated sides. Also, the values of the stsv (side-to-side variability) ratio showed dysfunction affected both sides (Figure 2). Our results are in opposition to other papers, in which authors described that diaphragm movement was strongly impaired on the operated side. In the first paper, Spadaro et al. [16] assessed diaphragm motion 2 and 24 h after surgery, while in our study measurements were performed 3–5 days after surgery. On the basis of these findings, we can speculate that, initially, postoperative diaphragm dysfunction can be one-sided, but over time turns into both-side impairment. However, further studies precisely monitoring postoperative diaphragm function day by day are needed to confirm our hypothesis. In the case of the second paper, the dissimilarities of the findings can be a result of different study populations. In Takazura’s [14] study, almost 70% of patients had undergone upper-lobe resection and bilobectomy. While our clinical group consisted of a similar patient number for each resected lobe, we excluded patients after bilobectomy and segmentectomy. However, according to data presented in Table 4, the left and right upper-lobe resection was associated with slightly greater impairment of hemidiaphragm strength and contractility on the operated side. Sekine et al. [15] demonstrated that lobectomy of the lower portion resulted in better residual lung function than lobectomy of the upper portion in lung cancer patients with chronic obstructive pulmonary disease.

At this moment, we do not know whether the described diaphragm dysfunctions are permanent or transient phenomena, and if they are transient, we also do not know how long the diaphragm function takes to return to physiological values. There are a few previous studies that reported faster recovery of respiratory muscle strength measured as Maximal Inspiratory Pressure [20,23,24]. However, all of these studies have some limitations due to the fact MIP is a general index not directly used to singularly assess the diaphragm muscle, thereby not allowing the evaluation of the hemidiaphragm and not differentiating dysfunction due to the side of operation. Takazura [14] showed that diaphragmatic motion on the operated side was significantly decreased, whilst on the non-operated side it was significantly increased as a compensatory mechanism. Maeda et al. [13] showed an increase in intercostal muscle recruitment after pulmonary resection. Perhaps, similar mechanisms can be encountered in the case of MIP improvement. For this reason, other studies should be designed to evaluate diaphragm function after thoracic surgeries in a long-term perspective. However, we know that the reduction in the DTF index emphasizes the necessity of the use of pulmonary rehabilitation based on respiratory exercises after thoracic surgery procedures to restore the proper function of the diaphragm.

In summary, the results of our study again emphasize the lower invasiveness of the lobectomy via VATS versus conventional thoracotomy. The actual prevalence of post-surgery diaphragmatic dysfunction may be underestimated as many patients may have no noticeable and specific symptoms, as well as the fact that diaphragm ultrasound is not a gold standard in postoperative care [25]. The causes of diaphragmatic dysfunction remain incompletely understood. Several mechanisms including changes in reflexogenic inhibition of phrenic nerve activation, pain and direct diaphragmatic muscle injury have been proposed [26,27]. However, according to the latest study, the reduction in respiratory muscle function after operation is not affected by postoperative pain alone because pain relief by epidural anesthesia did not reduce diaphragm dysfunction [28,29]. Postoperative sedation and analgesia administered intravenously also did not influence postoperative diaphragm dysfunction [30]. In our study, the surgeons did not use electrocoagulation in the area of the phrenic nerve during lobectomy, which could cause damage or even temporary paralysis of the phrenic nerve. The perioperative diaphragm damage was also not reported. 

## 5. Conclusions

Based on the findings presented here, one may draw several clinically relevant conclusions regarding the purposes defined at the beginning of the study.

1. Generally, greater diaphragm impairment was observed after lobectomy via conventional thoracotomy compared to VATS.
2. Inspiratory Thickness, Diaphragm Thickness Fraction (DTF) reflecting the magnitude of diaphragm effort and the Diaphragm Thickening Ratio (DTR) reflecting diaphragm muscle strength were significantly reduced after lobe resection in both groups, but the percentage of deterioration was greater after thoracotomy compared to VATS.
3. The percentage of hemidiaphragm paralysis was significantly higher after thoracotomy compared to VATS. Other types of diaphragm dysfunction (atrophy, weakness) were at similar levels after surgery compared to preoperative data.
4. The degree of diaphragm impairment differed according to the location of the resected lobe. Left-upper and right-upper resection was associated with greater diaphragm impairment compared to the case of resection of other lobes

## Figures and Tables

**Table 1:** Baseline demographic and clinical data.

Variables	VATS	OT	Control Group	*p*-Value
Number of subjects	30	29	28	-
Age:				
Mean (years)	63.44 ± 12.02	65.07 ± 11.99	59.13 ± 11.46	0.334
Range (min–max)	(42–73)	(45–78)	(35–69)	-
Height (cm)	168.15 ± 12.07	172.99 ± 15.26	173.01 ± 14.35	
Weight (kg)	76.95 ± 14.57	73.91 ± 16.18	75.64 ± 15.87	0.633
BMI (kg/m^2^)	27.59 ± 8.36	25.61 ± 9.19	25.84 ± 10.13	0.577
Gender:				0.213
Male	18 (60.0%)	15 (51.7%)	13 (46.4%)	
Female	12 (40.0%)	14 (48.3%)	15 (53.6%)	
Involved side:				0.426
Left	15 (50.0%)	13 (44.8%)	-	
Right	15 (50.0%)	16 (55.2%)	-	
Control side:				
Left	-	-	14 (50.0%)	1.00
Right	-	-	14 (50.0%)	
Resected lobe:				0.119
Right-upper	9 (30.0%)	7 (24.1%)	-	
Right-middle	2 (6.7%)	2 (6.9%)	-	
Right-lower	6 (20.0%)	6 (20.7%)	-	
Left-upper	7 (23.3%)	6 (20.7%)	-	
Left-lower	6 (20.0%)	8 (27.6%)	-	
Complete radical				1.00
Lymphadenectomy:				
Yes	30 (100.0%)	30 (100.0%)	-	
No	0 (0.0%)	0 (0.0%)	-	
Lymph Nodes resection:				0.144
LNs 4	9 (30.0%)	7 (24.1%)	-	
LNs 5	7 (23.3%)	6 (20.7%)	-	
LNs 6	0 (0.0%)	0 (0.0%)	-	
LNs 7	30 (100%)	29 (100%)	-	
LNs 8	12 (40.0%)	14 (48.2%)	-	
LNs 9	12 (40.0%)	14 (48.2%)	-	
LNs 10	30 (100%)	29 (100%)	-	
LNs 11	30 (100%)	29 (100%)	-	

Abbreviations: LNs—lymph nodes.

**Table 2:** Preoperative (pre) and postoperative (post) descriptive statistics of diaphragm muscle thickness.

Variables			Th Ins Left	Th Ins Right	Th Exp Left	Th Exp Right
Left Side Operated	VATS	Pre	2.97 ± 0.47 (2.73–3.21)	2.90 ± 0.54 (2.65–3.06)	2.14 ± 0.39 (1.94–2.34)	2.14 ± 0.41 (1.93–2.35)
		Post	2.59 ± 0.40 (2.35–2.76)	2.57 ± 0.46 (2.31–2.78)	1.95 ± 0.40 (1.74–2.15)	1.98 ± 0.39 (1.78–2.18)
		*p*-value	0.12	0.019	0.113	0.126
		Δ	0.38 *	0.33 *	0.19	0.16
		% Δ	12.79	11.37 *	8.87 *	7.47
	OT	Pre	3.11 ± 0.65 (2.74–3.38)	3.05 ± 0.68 (2.67–3.41)	2.32 ± 0.61 (1.98–2.66)	2.32 ± 0.62 (1.98–2.67)
		Post	2.61 ± 0.63 (2.23–2.98)	2.59 ± 0.62 (2.24–2.99)	2.23 ± 0.64 (1.85–2.61)	2.22 ± 0.65 (1.83–2.60)
		*p*-value	<0.001	<0.001	0.531	0.506
		Δ	0.50 *	0.46 *	0.09	0.10
		% Δ	16.07 *	15.08 *	3.87 *	4.31
Right Side Operated	VATS	Pre	2.88 ± 0.31 (2.72–3.03)	2.95 ±0.32 (2.79–3.11)	2.14 ± 0.38 (1.95–2.33)	2.14 ± 0.36 (1.96–2.33)
		Post	2.61 ± 0.45 (2.38–2.84)	2.68 ± 0.41 (2.47–2.89)	1.96 ± 0.47 (1.72–2.19)	1.98 ± 0.47 (1.74–2.22)
		*p*-value	0.024	0.021	0.139	0.131
		Δ	0.27 *	0.27 *	0.18	0.17
		% Δ	9.37 *	9.15 *	8.41	7.94
	OT	Pre	3.09 ± 0.45 (2.86–3.28)	3.07 ± 0.46 (2.85–3.19)	2.10 ± 0.47 (1.88–2.32)	2.10 ± 0.45 (1.88–2.31)
		Post	2.55 ± 0.49 (2.32–2.79)	2.56 ± 0.50 (2.31–2.78)	2.03 ± 0.42 (1.83–2.23)	2.05 ± 0.41 (1.85–2.24)
		*p*-value	<0.001	<0.001	0.445	0.469
		Δ	0.54 ***	0.51 ***	0.07	0.05
		% Δ	17.47 ***	16.61 ***	3.33	2.38

Notes: data are presented as mean ± standard deviation (95% Confidence Interval). Difference levels between VATS and OT groups are marked as follows: * *p* < 0.05, *** *p* < 0.001.

**Table 3:** Preoperative (pre) and postoperative (post) descriptive statistics of diaphragm function indices.

Variables			DTF Left	DTF Right	DTR Left	DTR Right
Left Side Operated	VATS	Pre	36.24 ± 23.51 (24.34–48.14)	32.60 ± 20.59 (22.18–43.02)	1.36 ± 0.24 (1.23–1.49)	1.32 ± 0.20 (1.22–1.43)
		Post	25.89 ± 18.00 (16.78–35.00)	26.82 ± 16.28 (18.58–35.06)	1.25 ± 0.18 (1.16–1.35)	1.27 ± 0.16 (1.18–1.35)
		*p* value	<0.001	<0.001	0.492	0.786
		Δ	10.35 ***	5.78 *	0.11	0.05
		% Δ	28.55 ***	17.73 ***	8.08	3.78
	OT	Pre	36.45 ± 22.71 (23.60–49.31)	33.43 ± 23.57 (20.09–46.77)	1.36 ± 0.22 (1.24–1.48)	1.33 ± 0.23 (1.20–1.46)
		Post	18.41 ± 16.01 (8.95–27.88)	19.59 ± 16.18 (10.02–29.15)	1.18 ± 0.16 (1.09–1.27)	1.19 ± 0.16 (1.10–1.29)
		*p*-value	<0.001	<0.001	0.037	0.048
		Δ	18.04 ***	13.84 ***	0.18	0.14
		% Δ	49.41 ***	41.39 ***	13.23	10.52
Right Side Operated	VATS	Pre	42.20 ± 18.07 (27.06–45.35)	45.57 ± 24.00 (28.42–52.72	1.38 ± 0.18 (1.27–1.46)	1.41 ± 0.24 (1.28–1.52)
		Post	35.18 ± 23.00 (25.54–48.82)	35.66 ± 19.36 (18.67–33.53)	1.29 ± 0.23 (1.25–1.48)	1.27 ± 0.47 (1.19–1.67)
		*p*-value	0.022	0.037	0.507	0.046
		Δ	7.02	9.91	0.09	0.14
		% Δ	16.63 ***	21.74 ***	6.52	9.92
	OT	Pre	41.11 ± 23.12 (25.36–56.85)	45.01 ± 27.04 (30.32–61.71)	1.51 ± 0.34 (1.35–1.67)	1.51 ± 0.33 (1.35–1.66)
		Post	27.85 ± 21.30 (17.72–37.97)	25.00 ± 17.94 (16.47–33.53)	1.27 ± 0.21 (1.17–1.38)	1.25 ± 0.18 (1.16–1.34)
		*p*-value	<0.001	<0.001	0.008	0.003
		Δ	13.26 ***	20.01 ***	0.24	0.26
		% Δ	32.25 ***	44.45 ***	15.89	17.21

Notes: data are presented as mean ± standard deviation (95% Confidence Interval). Differences levels between VATS and OT groups are marked as follows: * *p* < 0.05, *** *p* < 0.001.

**Table 4:** Percentage values of Δ indexes and level of differences between VATS and OT groups.

Variables		ΔTh Ins Left	ΔTh Ins Right	ΔTh Exp Left	ΔTh Exp Right	ΔDTF Left	ΔDTF Right	ΔDTR Left	ΔDTR Right
Upper-right Resection	VATS	15.85 †	17.58	6.99 *	7.43 *	26.39	30.72 ††	7.47	9.23
	OT	18.01 †	19.24 †	3.19 *	3.26 *	30.23	34.06 ††	11.13	11.84
Upper-left Resection	VATS	14.69	13.85	12.26 *†	10.24 *†	33.45 †	23.61	10.13	8.77
	OT	17.67	15.26	3.32 *	3.27 *	35.27 ††	27.89 †	11.82	10.67
Lower-right Resection	VATS	6.23 †	15.12	6.74	6.37	22.24	17.44 ††	6.13	8.66
	OT	13.78	14.89	3.28	3.40	25.65	19.88 ††	9.27	10.18
Lower-left Resection	VATS	11.87	9.11	6.02	5.25	20.95 †	18.32	6.06	5.42
	OT	14.34	13.75	3.63	3.39	21.54 ††	19.37 †	9.88	9.31

Notes: differences levels between VATS and OT groups are marked as follows: * *p* < 0.05. Difference levels between upper and lower resection are marked as follows: † *p* < 0.05, †† *p* < 0.01.

## Data Availability

The data presented in this study are available upon request from the corresponding author.
